# Supplementary figures and images for: Transcriptomic and metabolomic analysis of autumn leaf color change in Fraxinus angustifolia
Source: PeerJ. 2023 May 12;11:e15319. doi: 10.7717/peerj.15319 (PMC10184661; doi:10.7717/peerj.15319)

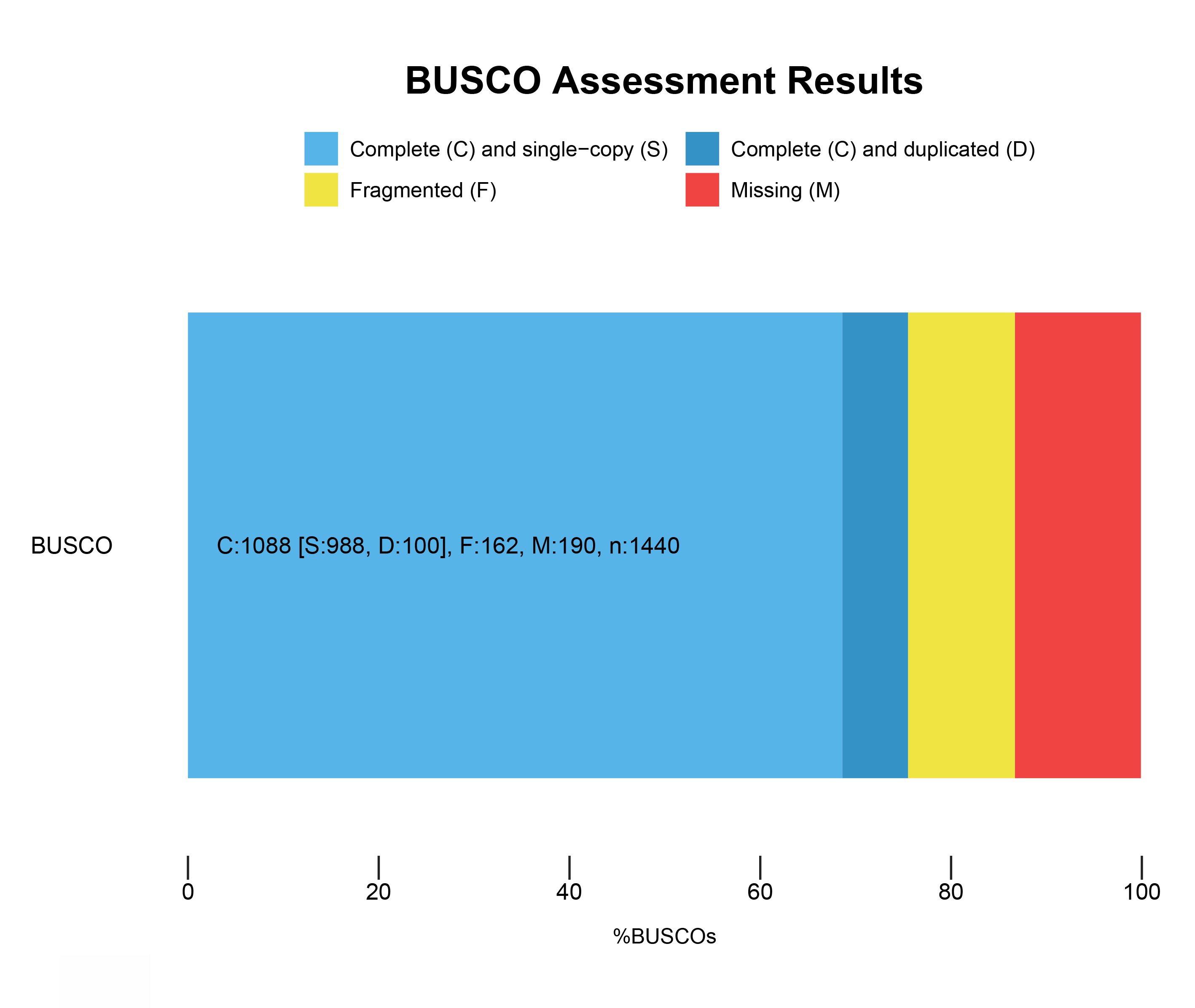

Supplement: Supplemental Information 1 [file peerj-11-15319-s001.jpg]

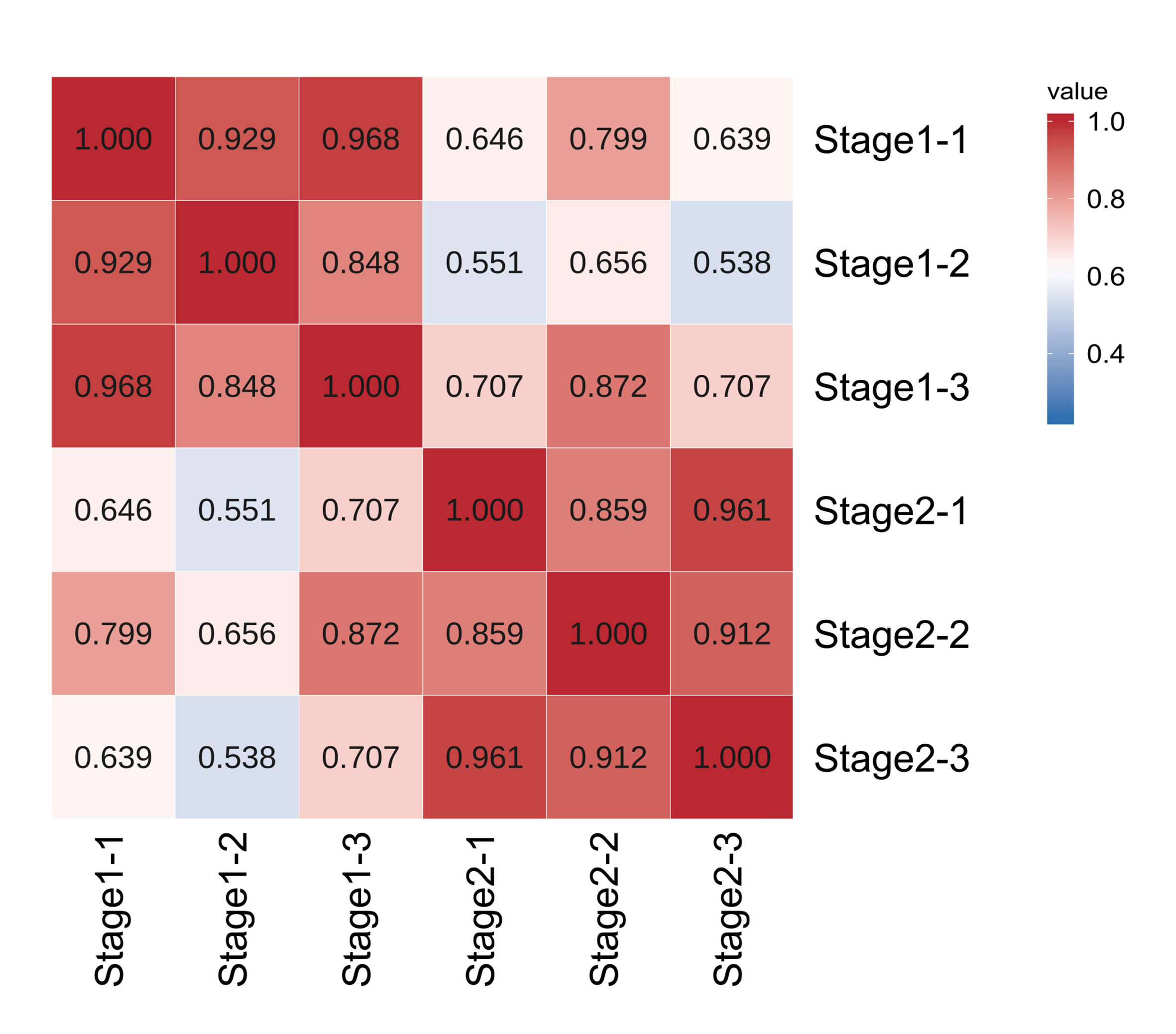

Supplement: Supplemental Information 2 [file peerj-11-15319-s002.jpg]

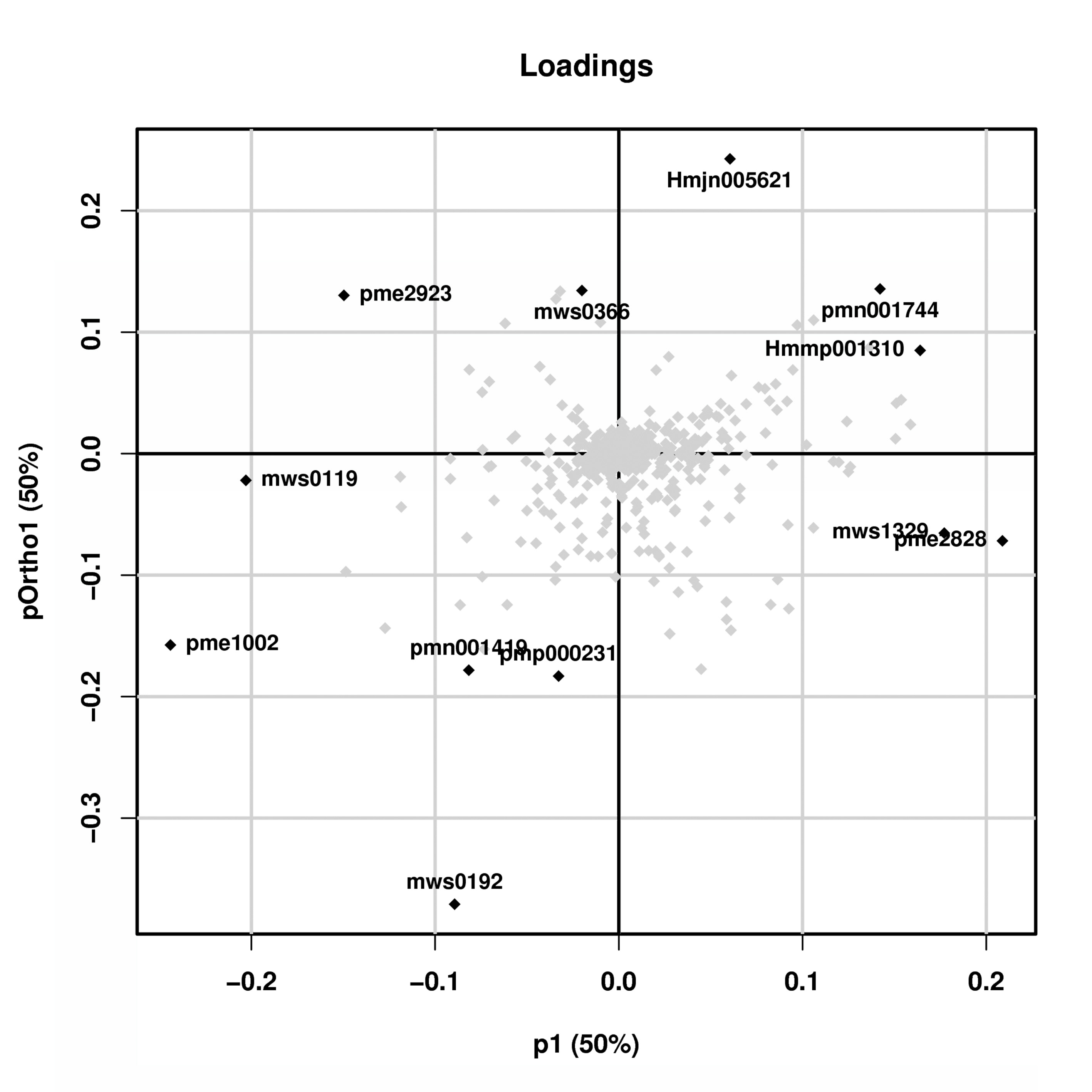

Supplement: Supplemental Information 3 [file peerj-11-15319-s003.jpg]

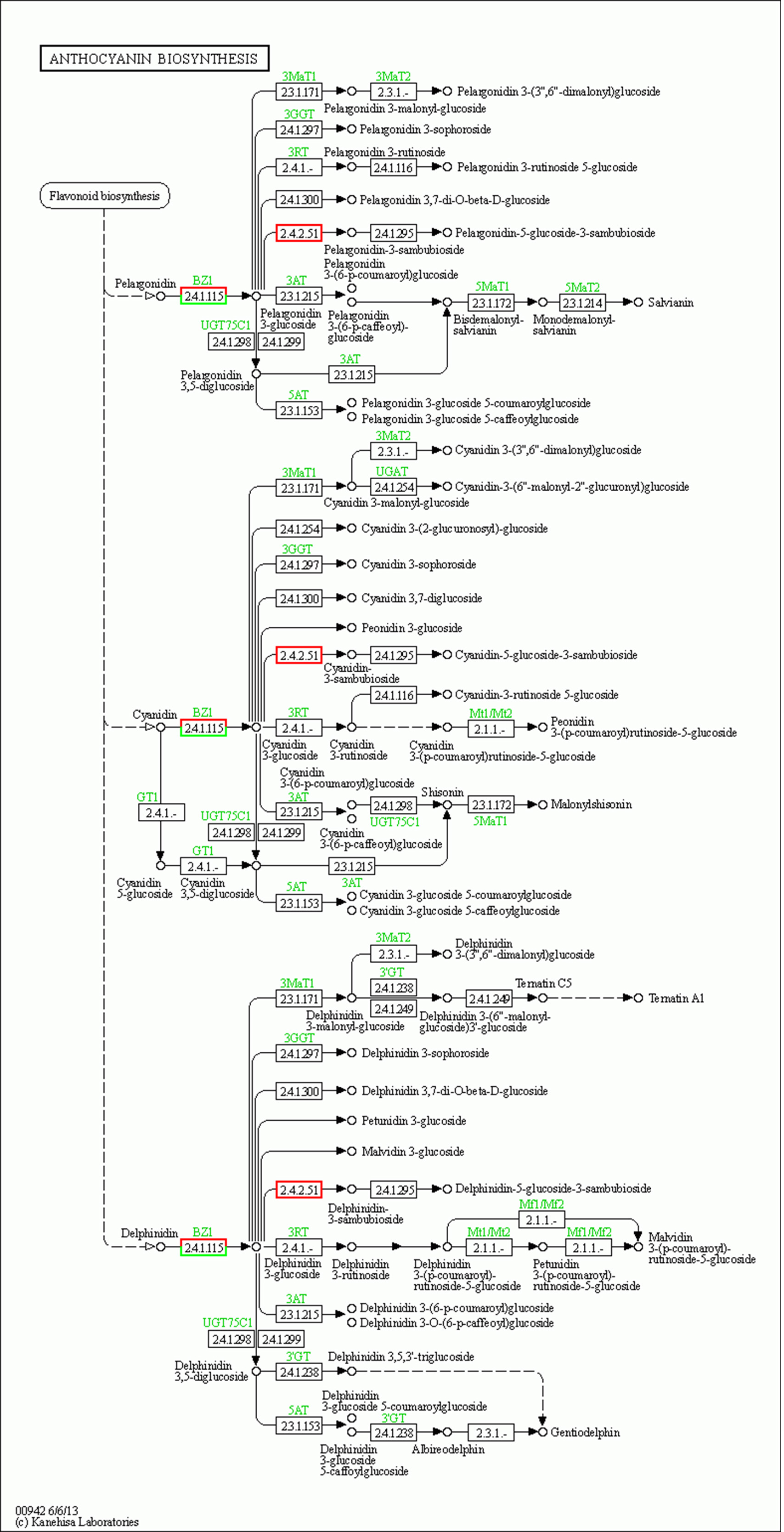

Supplement: Supplemental Information 4 [file peerj-11-15319-s004.jpg]
