# Supplementary material for: Transcriptomic and metabolomic analysis of autumn leaf color change in Fraxinus angustifolia
Source: PeerJ. 2023 May 12;11:e15319. doi: 10.7717/peerj.15319 (PMC10184661; doi:10.7717/peerj.15319)
Supplement: Supplemental Information 5 [file peerj-11-15319-s005.docx]

**Table** **S1.** Statistics of transcriptome clean data.

| Sample ID | Reads Number | Base Number | GC Content | %≥Q30 |
| --- | --- | --- | --- | --- |
| Stage1-1 | 7,049,775,900 | 6,969,833,211 | 45.32% | 93.73% |
| Stage1-2 | 5,791,585,200 | 5,735,993,483 | 45.44% | 95.09% |
| Stage1-3 | 6,418,271,400 | 6,377,888,504 | 45.27% | 94.51% |
| Stage2-1 | 10,874,368,200 | 10,788,329,801 | 45.21% | 94.30% |
| Stage2-2 | 6,760,174,800 | 6,719,141,845 | 44.98% | 95.20% |
| Stage2-3 | 6,909,533,100 | 6,845,572,783 | 45.12% | 93.86% |
